# Antimicrobial susceptibility of staphylococci from bovine milk samples in routine microbiological mastitis analysis in Finland

**DOI:** 10.3389/fvets.2023.1235417

**Published:** 2023-08-17

**Authors:** Suvi Taponen, Heikki-Tapio Tölli, Päivi J. Rajala-Schultz

**Affiliations:** Department of Production Animal Medicine, Faculty of Veterinary Medicine, University of Helsinki, Saarentaus, Finland

**Keywords:** *Staphylococcus aureus*, non-aureus staphylococci, NAS, bovine mastitis, antimicrobial resistance, penicillin, *blaZ*, *mec*

## Abstract

The most frequent reason for antimicrobial use in dairy herds is mastitis and knowledge about mastitis-causing pathogens and their antimicrobial susceptibility should guide treatment decisions. The overall objective of this study was to assess antimicrobial resistance (AMR) of staphylococci in mastitic milk samples in Finland. MALDI-ToF MS identified a total of 504 *Staphylococcus* isolates (260 *S. aureus* and 244 non-aureus staphylococci, NAS) originating from bovine mastitic milk samples. Phenotypic susceptibility against cefoxitin, ceftiofur, enrofloxacin, gentamycin, oxacillin, penicillin, and tetracycline was evaluated by disk diffusion method and the presence of *blaZ*, *mecA*, and *mecC* genes investigated by PCR. Nitrocefin test assessed these isolates’ beta-lactamase production. The most common NAS species were *S. simulans, S. epidermidis, S. chromogenes,* and *S. haemolyticus.* In total, 26.6% of the isolates (18.5% of *S. aureus* and 35.2% of all NAS) carried the *blaZ* gene. Penicillin resistance, based on disk diffusion, was lower: 18.8% of all the isolates (9.3% of *S. aureus* and 28.9% of all NAS) were resistant. Based on the nitrocefin test, 21.5% of the isolates produced beta-lactamase (11.6% of *S. aureus* and 32.0% of all NAS). Between the *Staphylococcus* species, the proportion of penicillin-resistant isolates varied, being lowest in *S. simulans* and highest in *S. epidermidis.* Resistance to antimicrobials other than penicillin was rare. Of the eight NAS isolates carrying the *mecA* gene, six were *S. epidermidis*. One *S. aureus* isolate carried the *mecC* gene. Agreement beyond chance, assessed by kappa coefficient, between phenotypic and genotypic resistance tests, was moderate to substantial. Some phenotypically penicillin-susceptible staphylococci carried the *blaZ* gene but isolates without *blaZ* or *mec* genes rarely exhibited resistance, suggesting that the more reliable treatment choice may depend upon genotypic AMR testing. Our results support earlier findings that penicillin resistance is the only significant form of antimicrobial resistance among mastitis-causing staphylococci in Finland.

## Introduction

The most frequent reason for antimicrobial use in dairy herds is mastitis (1). Microbiological diagnosis and knowledge of the antimicrobial susceptibility of pathogens form the basis for effective treatment of intramammary infections. Antimicrobial resistance has been a growing global concern during recent decades (2, 3), with solutions to combat the problem requiring joint efforts within both human and veterinary medicine, in a true One Health spirit (4, 5). Antimicrobial use can lead to resistant strains, and resistance mechanisms may be transmitted from one bacterial species or strain to another through mobile genetic elements (6). Monitoring the antimicrobial resistance of mastitis-causing pathogens is important for ensuring the continued availability of efficacious treatments.

In Finland, prudent antimicrobial use guidelines for mastitis therapy are followed: Bacteriologic analysis of milk samples before initiation of antimicrobial treatment is a common practice and penicillin is the drug of choice in mastitis caused by Gram-positive bacteria (7). Additionally, selective dry cow therapy (SDCT) has always been implemented, with only approximately one-fourth of cows receiving antibiotic dry cow treatment at the end of lactation (8). Since 2010, a mastitis diagnosis in Finland has relied predominantly on PCR methodology after the main dairy co-operative in the country switched to PCR in its mastitis laboratory. Later, also a private veterinary diagnostic laboratory and the clinical laboratory at the Department of Production Animal Medicine at the University of Helsinki adopted this technology. Veterinarians occasionally use bacterial culture and selective agar plates in their clinic laboratories to achieve a quick bacterial diagnosis during weekends and on-call work. The commercial PCR kit (PathoProof™ Complete-16 PCR Assay, Thermo Fisher Scientific, Waltham, MA, United States) used in these diagnostic laboratories targets the 15 main mastitis pathogens plus the staphylococcal *blaZ* gene which codes for beta-lactamase production. Russi et al. (9) reported a discrepancy between a finding of the *blaZ* gene and phenotypic penicillin resistance, a phenomenon also observable in the clinical laboratory of Production Animal Hospital, Faculty of Veterinary Medicine, University of Helsinki. After adopting the PCR methodology, the laboratory has used both PCR and phenotypic beta-lactamase production tests for determining the antimicrobial susceptibility of staphylococci. Discrepancies in results make treatment decisions challenging.

*Staphylococcus* spp. are the most prevalent causal agents of intramammary infections in Finland, constituting approximately 64% of all pathogens isolated from IMIs (10). Resistance of staphylococci against antimicrobials other than beta-lactams is infrequently monitored in Finland, since penicillin is the drug of choice for treatment of infections caused by Gram-positive cocci. In routine diagnostics, staphylococcal species, except for *Staphylococcus aureus*, are not identified beyond genus level in the PCR-based mastitis diagnostics, although some studies have shown differences between the species in antimicrobial susceptibility (11, 12).

Methicillin resistance coded by the *mecA* and *mecC* genes is of great concern in both human and veterinary medicine, due to the zoonotic nature and role of *S. aureus* in both community-acquired and nosocomial infections (13, 14). Although methicillin resistance is rare in staphylococci isolated from bovine mastitis (12, 15, 16), methicillin-resistant *S. aureus* (MRSA) have appeared rather commonly in swine production (17, 18), in horses, and in companion animals (19–21).

The main objective of this study was to evaluate the antimicrobial susceptibility of mastitis-causing staphylococci against those antimicrobial agents most frequently used in treatment of bovine mastitis and other infectious diseases of cattle. The other objectives were to estimate the prevalence of methicillin resistance, and to compare the genotypic and phenotypic beta-lactamase resistance in these staphylococci.

## Materials and methods

### Milk samples

The study material comprised milk samples submitted for bacteriologic analysis to the mastitis laboratory of the main Finnish dairy company Valio Ltd. The company is a farmer-owned cooperative with members across the entire country; it processes approximately 80% of all the milk produced in Finland. Finnish dairy farmers and veterinarians frequently send milk samples from clinical mastitis cases to that laboratory for bacteriologic analysis. They typically receive results electronically the next day by text message, by email, or through a herd-health program of the dairy cooperative. Additionally, milk samples from subclinically infected, high-SCC quarters are often collected before treatment, e.g., at dry-off, to ensure selection of the most appropriate and effective treatment. For its mastitis diagnosis, the Valio laboratory uses PathoProof™ Complete-16 PCR Assay (Thermo Fisher Scientific), which targets 15 mastitis-causing microbial species and the staphylococcal *blaZ* gene. Each week between August and November 2017, the laboratory personnel saved and froze milk samples which according to the PCR assay contained substantial amounts of the DNA of *S. aureus* (cycle threshold, Ct-value ≤30.0) or non-aureus staphylococci, NAS (Ct-value ≤31.0) for later culturing and antimicrobial susceptibility testing.

Assuming 20% of *S. aureus* to be penicillin resistant (17), the Epitools calculator, using 80% power and 5% significance level, provided a sample size of 246 *S. aureus* isolates to estimate the proportion of penicillin-resistant *S. aureus* with 5% precision (22). Our goal was to collect the same number of NAS isolates. Only one isolate of any staphylococcal species detected in a herd was included in the data analysis, to ensure the observations’ epidemiologic independence. If the staphylococcal species, however, differed from each other, multiple samples and isolates may have been included from the same cow or the same farm,

### Isolate culture and speciation

In the laboratory of the Department of Production Animal Medicine, Faculty of Veterinary Medicine, University of Helsinki, the milk samples were thawed, and 10 μL of milk was streaked on sheep-blood agar plates (Oxoid, Thermo Fisher) and incubated for 24 h at +37°C. Potential staphylococcal colonies were identified based on colony morphology and hemolysis and then MALDI-ToF MS (Bruker Maldi Biotyper, Bruker Daltonics Scandinavia AB, Solna, Sweden) determined the species by the direct transfer method (23). The PCR assay identifies NAS only at group level. If, however, MALDI-ToF MS identified more than one NAS species in a sample, isolates of differing species were considered for the study. Similarly, if a sample contained both *S. aureus* and NAS, both were considered in the study. However, samples positive for differing species and the *blaZ* gene were excluded from the study, because it is not possible to know which species carried the *blaZ* gene. Samples with >2 different colony types were excluded from the study.

### Susceptibility testing

The antimicrobial susceptibility to cefoxitin, ceftiofur, enrofloxacin, gentamycin, oxacillin, penicillin, and tetracycline was evaluated using the disk diffusion method. The disks (Oxoid, Thermo Fisher) contained cefoxitin 30 μg, ceftiofur 30 μg, enrofloxacin 5 μg, gentamycin 10 μg, oxacillin 1 μg, penicillin 10 μg, and tetracycline 30 μg. For the cut-off/breakpoint values used and their sources see Table 1. The epidemiologic cut-off (ECOFF) values for susceptibility of staphylococcal species were used when available (24). For ceftiofur, the values were CLSI clinical breakpoints for mastitis in cattle (25). For benzylpenicillin, epidemiologic cut-off values exist only for 1 μg disks, whereas we used 10 μg disks, and thus used a CLSI clinical breakpoint value for human staphylococci. Beta-lactamase production of the isolates was tested by use of liquid nitrocefin prepared from nitrocefin powder as recommended by the manufacturer (Nitrocefin Solution, Oxoid, Thermo Fisher).

**Table 1:** Epidemiologic cut-off and clinical breakpoint values (mm) for categorizing bacterial isolates as susceptible or resistant in disk diffusion test.

Antimicrobial	Disk content	Susceptible (mm)		Reference
		*Staphylococcus aureus*	NAS	
Cefoxitin	30 μg	≥22	≥25	ECOFF, EUCAST 2021
Ceftiofur	30 μg	≥21	≥21	Clinical breakpoint, *S. aureus* mastitis in cattle, CLSI 2008
Enrofloxacin	5 μg	>23	>23	Clinical breakpoint, *Staphylococcus* skin and soft tissue infections in dogs, CLSI 2008
Gentamycin	10 μg	≥18	≥22	ECOFF, EUCAST 2021
Oxacillin	1 μg	≥20	≥20	Clinical EUCAST breakpoint for screening methicillin resistance in *S. pseudintermedius* and *S. schleiferi,* EUCAST 2021
Penicillin	10 IU	≥29	≥29	Clinical breakpoint, human *Staphylococcus* infections, CLSI 2008
Tetracycline	30 μg	≥22	≥22	ECOFF, EUCAST 2021

### PCR analyses of *mecA* and *mecC* genes

The presence of the *mecA* or *mecC* genes was analyzed by PCR in 158 isolates with oxacillin inhibition zone ≤20 mm or cefoxitin inhibition zone ≤28 mm, to ensure detection of all *mecA-* or *mecC-*positive isolates. The PCR for detection of *mecA* or *mecC* was performed as described in DTU (Technical University of Denmark) Food protocols recommended by the EURL-AR (26). The primers for the analyses were *mecA1*(P4) 5′-TCC AGA TTA CAA CTT CAC CAG G-3′, *mecA2*(P7) 5′-CCA CTT CAT ATC TTC TAA CG-3′, *mecC1*(MultiFP) 5′-GAA AAA AAG GCT TAG AAC GCC TC-3′, and *mecC2*(MultiRP) 5′-GAA GAT CTT TTC CGT TTT CAG C-3′ (Metabion international AG, Steinkirchen, Germany). The *mecA*-positive *S. aureus* ATCC 43300 (162 bp) and the *mecC*-positive *S. aureus* CCUG 63582 (138 bp) served as control strains. Distilled water served as the negative control.

### Statistical analyses

Descriptive statistics (median, minimum, and maximum inhibitory zone diameters and proportions of isolates resistant for each antimicrobial drug) were calculated separately for *S. aureus*, for the four most prevalent NAS species, and for the other NAS species together as a group. Proportions of the bacteria carrying *blaZ* or *mec* genes were calculated. Agreement beyond chance between phenotypic (based on a disk diffusion method and nitrocefin test) and genotypic (carriage of *bla*Z gene) resistance to penicillin was assessed by kappa coefficient. Statistical analyses were conducted with the Statistical Analysis System, v. 9.4 (SAS Inst Inc., Cary, NC, United States) and Epitools Calculator (22).

## Results

The antimicrobial susceptibility of 504 isolates was evaluated and included in the data analysis. These isolates originated from 497 quarter milk samples from 466 cows in 396 herds. Of these isolates, 260 were *S. aureus*, and 244 belonged to the NAS group. Altogether, 21 differing NAS species were detectable, including six isolates from three species previously included in the genus *Staphylococcus*: *S. lentus* (2), *S. sciuri* (3), and *S. vitulinus* (1), which recently have been reassigned to a novel genus, *Mammaliicoccus* (27). The most common NAS species were *S. simulans*, *S. epidermidis*, *S. chromogenes*, and *S. haemolyticus*, these accounting for approximately 74% (180/244) of all detectable NAS (Table 2). Isolates identified as *S. haemolyticus* may have included some isolates of a novel species, *S. borealis*, because MALDI-ToF MS does not differentiate between them (28). Of 28 samples with more than one staphylococcal species, six each contained *S. aureus* and a NAS species, and two had three different NAS species; the rest, 20 samples, each had two different NAS species. Of the 28 samples, in 21, the *blaZ* gene was also detectable, and these samples were excluded, because—based on the commercial PCR assay used—distinguishing which bacterial species carried the *blaZ* gene was impossible. Five *blaZ*-negative samples contained two different NAS species, one contained *S. aureus* and a NAS, and one sample had three different NAS species. All other samples had only one species each.

**Table 2:** Percentages of isolates carrying the *blaZ* gene encoding beta-lactamase production, and zone-inhibition diameters for penicillin in the most common staphylococcal species causing intramammary infections in dairy cows.

*Staphylococcus* species	*n*	% carrying *blaZ*	Inhibition zone diameters in mm: median (min – max); % resistant to penicillin^2^	% producing beta-lactamase^3^	% carrying *mec*^4^
*S. aureus*	260	18.5	46 (13–56); 9.3%	11.6	0.4%
*S. chromogenes*	37	29.7	41 (15–49); 21.6%	18.9	0%
*S. epidermidis*	49	79.6	24 (16–53); 70.2%	63.3	12.2%
*S. haemolyticus*	37	43.2	41 (6–48); 35.1%	35.1	2.7%
*S. simulans*	57	15.8	42 (18–49); 5.3%	5.3	0%
Other NAS^1^	64	17.2	40 (18–48); 36.1%	37.5	1.6%
All isolates	504	26.6	18.8%	21.5	1.8%

1 Fewer than 20 isolates of each of the following species detectable (*n* in parenthesis): *S. agnetis*/hyicus (15), *S. capitis* (8), *S. xylosus* (7), *S. equorum* (6), *S. kloosii* (5), *S. pasteuri* (4), *S. cohnii* (3), *M. sciuri* (3), *S. warneri* (3), *M. lentus* (2), *S. succinus* (2), *S. auricularis* (1), *S. gallinarum* (1), *S. hominis* (1), *S. pettenkoferi* (1), *S. saprophyticus* (1), and *M. vitulinus* (1).

2 Clinical breakpoint, human *Staphylococcus* infections, CLSI 2008.

3 Based on nitrocefin test.

4 Only those isolates with oxacillin-inhibition zone ≤20 mm or cefoxitin-inhibition zone ≤28 analyzed for mecA or mecC genes. Numbers are percentages carrying mec of the total number of isolates.

### Resistance to beta-lactams

Based on the detection of the *blaZ* gene in the PathoProof™ Complete-16 assay, of all 504 staphylococcal isolates, 134 (26.6%), this constituting 8.5% of *S. aureus* and 35.2% of all NAS, carried the *blaZ* gene. These could thus be considered resistant to penicillin. Penicillin resistance was lower based on disk diffusion: Of all the isolates, 18.8% (9.3% of *S. aureus* and 28.9% of all NAS) were penicillin resistant.

**Figure 1:**
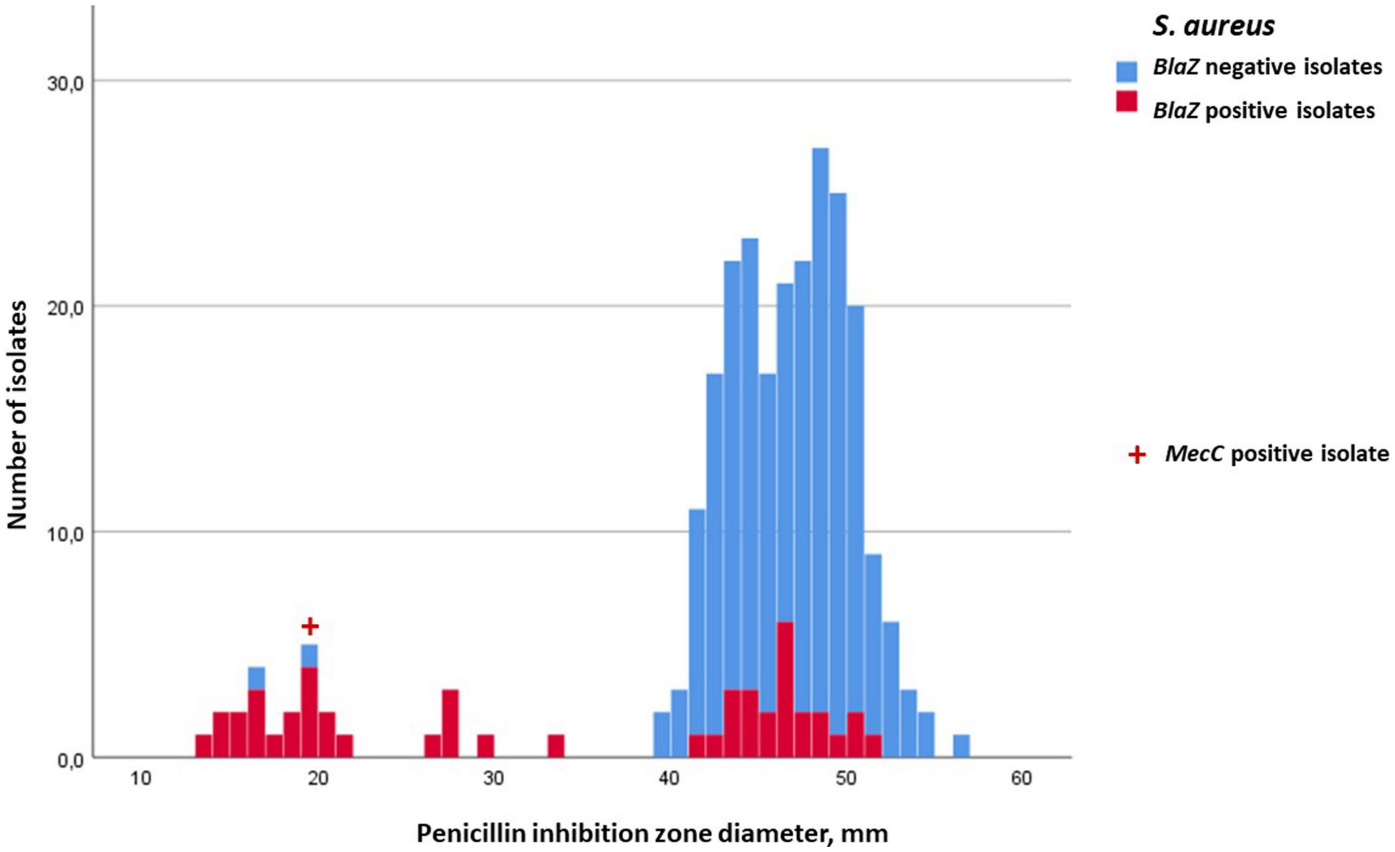
Frequency distribution of penicillin-inhibition zones for *Staphylococcus aureus*, occurrence of *blaZ* gene (no = blue; yes = red) and carriage of *mecC* gene (indicated with **+**). The cut-off for penicillin resistance in the disk diffusion test was 29 mm. No *S. aureus* carried *mecA* gene.

**Figure 2:**
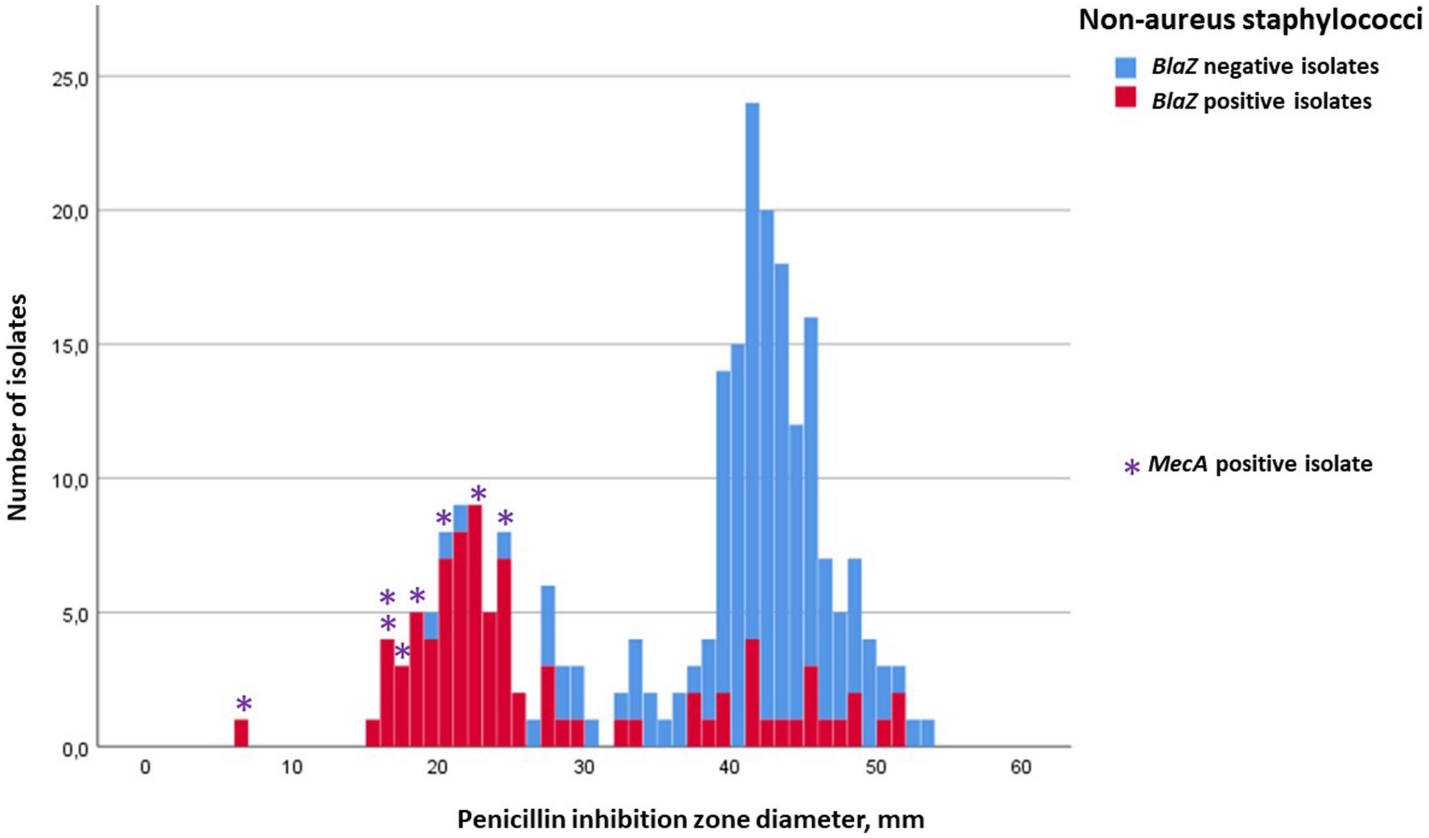
Frequency distribution of penicillin-inhibition zones for non-aureus staphylococci (NAS), occurrence of *blaZ* gene (no = blue; yes = red) and carriage of *mecA* gene (indicated with ^*^). The cut-off for penicillin resistance in the disk diffusion test was 29 mm. No NAS isolates carried *mecC* gene.

**Figure 3:**
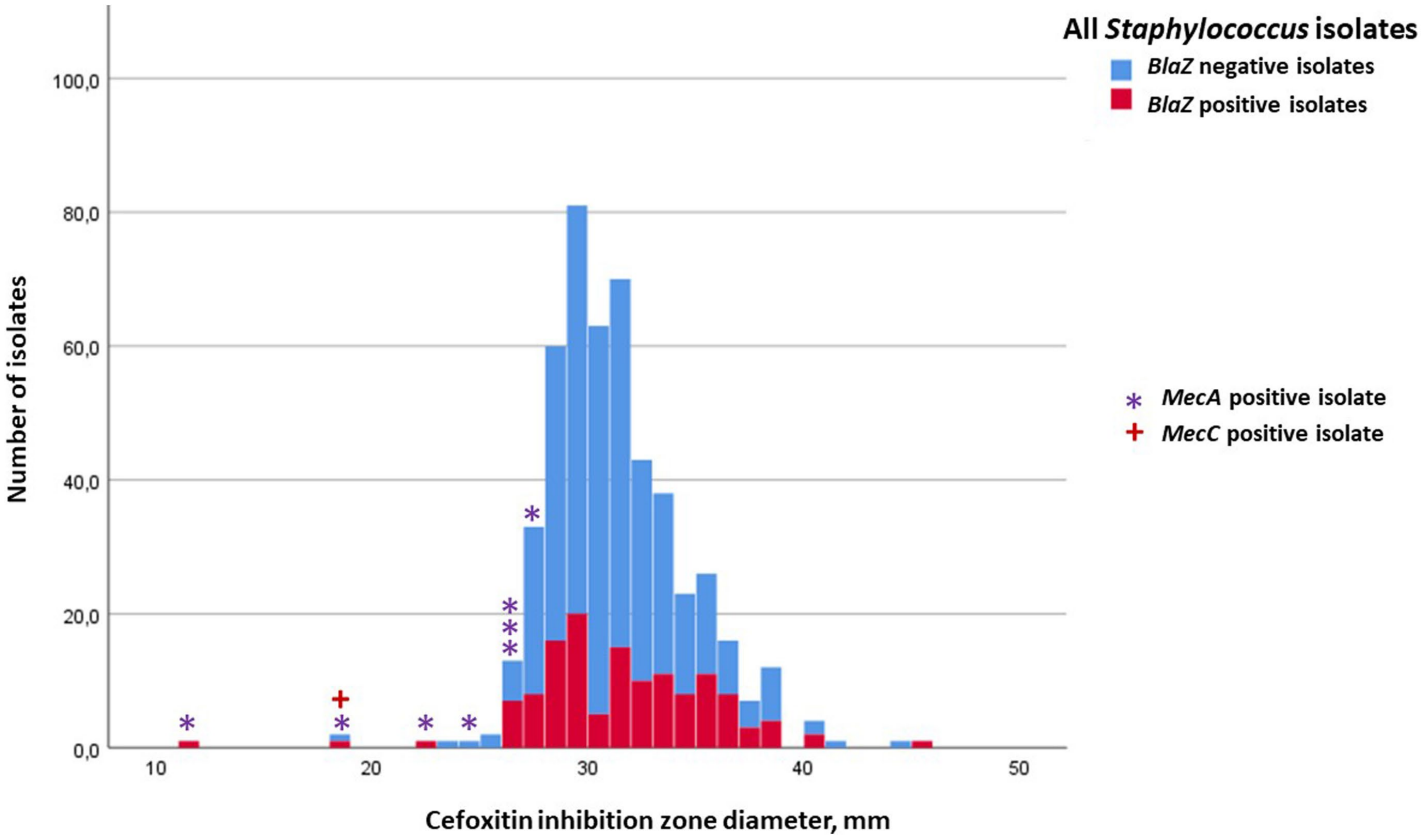
Frequency distribution of cefoxitin-inhibition zones for all staphylococcal isolates, occurrence of *blaZ* gene (no = blue; yes = red) and carriage of *mecA* (indicated with *) or *mecC* gene (indicated with **+**). The cut-off for cefoxitin resistance in the disk diffusion test was 22 mm for *S. aureus* and 25 mm for non-aureus staphylococci.

**Figure 4:**
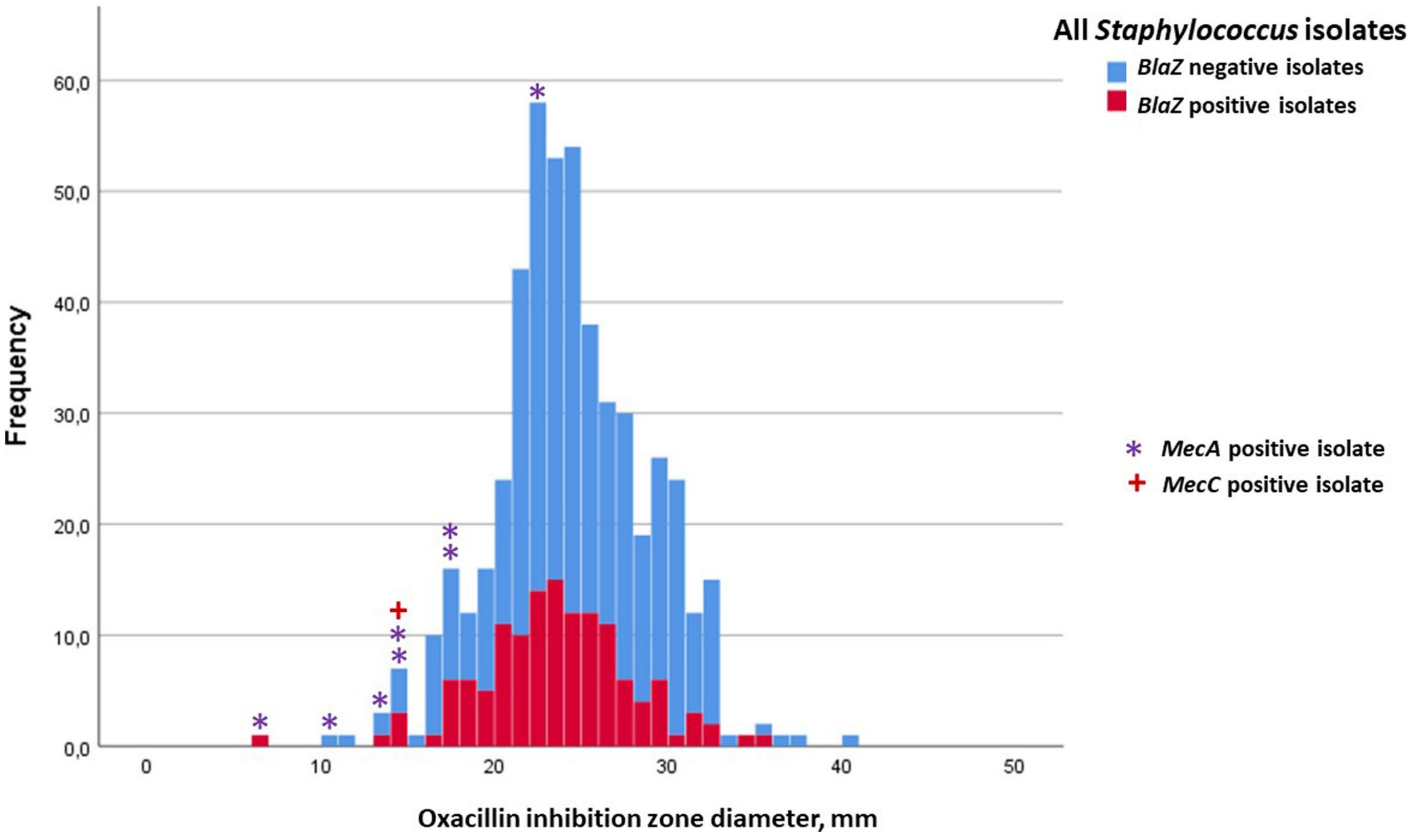
Frequency distribution of oxacillin inhibition zones for all staphylococcal isolates, occurrence of *blaZ* gene (no = blue; yes = red) and carriage of *mecA* (indicated with *) or *mecC* gene (indicated with **+**). The cut-off for oxacillin resistance in the disk diffusion test was 20 mm.

Inhibition-zone diameters (median, min, and max) of penicillin, cefoxitin, ceftiofur, and oxacillin for *S. aureus,* for the four most prevalent NAS species, and for the rest of the NAS group are in Tables 2, 3. Penicillin resistance based on disk diffusion testing was most common in *S. epidermidis*, of which 70.2% were resistant. The proportion of *blaZ*-positive *S. epidermidis* isolates was even higher, 79.6% (Table 2). Distribution of penicillin-inhibition zones, both for *S. aureus* and all NAS species, was bimodal, and the distribution of wild-type (isolates without acquired resistance) and resistant isolates was largely in agreement with the *blaZ*-gene occurrence (Figures 1, 2). In contrast to penicillin-inhibition zones, distribution of cefoxitin- and oxacillin-inhibition zones for all staphylococcal species was unimodal, and the *blaZ*-gene carriers were largely susceptible, especially to cefoxitin (Figures 3, 4). Based on the nitrocefin test, 21.6% of the isolates produced beta-lactamase (11.6% of *S. aureus* and 32.0% of all NAS; Table 2).

**Table 3:** Zone-inhibition diameters for cefoxitin, ceftiofur, and oxacillin in the most common staphylococcal species^1^ in dairy-cow intramammary infections.

		Inhibition-zone diameters in mm: median (min – max); % resistant^2^		
*Staphylococcus* species^1^	*N*	Cefoxitin	Ceftiofur	Oxacillin
*S. aureus*	260	30 (18–45); 0.3%	28 (16–36); 0.4%	24 (14–40); 10.1%
*S. chromogenes*	37	34 (26–44); 0%	28 (24–38); 0%	21 (17–31); 14.3%
*S. epidermidis*	49	35 (18–41); 4.6%	31 (22–36); 0%	26 (13–36); 10.6%
*S. haemolyticus*	37	29 (11–36); 2.1%	26 (6–30); 3.0%	22 (6–30); 10.6%
*S. simulans*	57	30 (27–35); 0%	28 (24–32); 0%	25 (22–30); 0%
Other NAS species^3^	64	32 (23–40); 0%	28 (18–34); 1.9%	21 (10–31); 46.1%
All isolates	504	1.0%	0.7%	13.6%

1 Species identification done with MALDI-TOF MS.

2 Percentage of isolates classified as non-susceptible (including resistant and intermediate), based on disk diffusion test using EUCAST epidemiologic cut-off values when available or CLSI clinical breakpoints (see text for details).

3 Fewer than 20 isolates of each of the following species detected (*n* in parenthesis): *S. hyicus* (17), *S. xylosus* (10), *S. capitis* (9), *S. equorum* (8), *S. kloosii* (5), *M. sciuri* (5), *S. cohnii* (4), *S. pasteuri* (4), *S. hominis* (3), *S. warneri* (3), *M. lentus* (2), *S. succinus* (2), *S. auricularis* (1), *S. caprae* (1), *S. condimenti* (1), *S. gallinarum* (1), *S. pettenkoferi* (1), *S. saprophyticus* (1), and *M. vitulinus* (1).

Only three isolates were resistant to ceftiofur (0.6%), one each of *S. aureus*, *S. haemolyticus*, and *M. lentus*. Five isolates (1.0%) were resistant to cefoxitin (two *S. epidermidis*, one *S. aureus*, one *S. haemolyticus*, and one *S. pettenkoferi*). Based on the threshold value for screening of methicillin resistance in *S. pseudintermedius* and *S. schleiferi* (24), 68 isolates (13.9%) were classifiable as resistant to oxacillin.

### *mecA* or *mecC* genes and agreement between different tests on beta-lactam resistance

In total, 158 staphylococcal isolates (84 *S. aureus* and 74 NAS) were tested for carriage of *mecA* and *mecC.* Eight of the NAS isolates carried *mecA*, six of these being *S. epidermidis*, one *S. haemolyticus*, and one *M. lentus* (Table 2). No *S. aureus* isolates carried *mecA*, but one was *mecC* positive. The penicillin-inhibition zones of the *mecA*-positive NAS isolates ranged from 6 to 24 mm, indicating penicillin resistance (Figure 2), but much variability existed in the cefoxitin- and oxacillin-inhibition zones. All *mec-*positive isolates were classified as resistant to penicillin in the disk diffusion test (Figures 1, 2). Of the eight *mecA*-positive isolates, four, and also the *mecC*-positive isolate, were resistant to cefoxitin (Figure 3). The *mec* carriage of the other four isolates would have remained undetected had the screening been based only on cefoxitin disk diffusion and the recommended cut-off values (24).

Of the nine *mec-*positive isolates, eight were resistant to oxacillin (Figure 4), and thus only one *mecA-*positive isolate remained undetected by oxacillin disk diffusion and the recommended cut-off value. On the other hand, of the 68 isolates classified as oxacillin resistant, only nine were *mec*-gene positive. Thus, 59 isolates were classified as oxacillin resistant based on disk diffusion but were *mec-*negative. Of the eight *mecA*-positive NAS isolates, seven also carried the *blaZ* gene, detectable by the PathoProof™ Complete-16 assay. The *mecC*-positive *S. aureus* isolate carried no *blaZ*, but its inhibition zone for penicillin was 19 mm, for ceftiofur 16 mm, for cefoxitin 18 mm, and for oxacillin 14 mm, all indicating its being resistant to all beta-lactam antimicrobials.

Of all isolates, 12 were *blaZ* PCR-negative but phenotypically resistant to penicillin (inhibition zones ≤28 mm). Two of these carried either the *mecA* or the *mecC* gene, but 10 were *mec*-gene negative. Conversely, 50 *blaZ*-positive isolates had penicillin inhibition zones ≥29 mm, ranging from 29 to 51 mm, indicating penicillin susceptibility. Five of these isolates had oxacillin inhibition zone diameters ≤19 mm, ranging from 14 to 18 mm (Figure 4).

Agreement beyond chance between phenotypic penicillin resistance based on the disk diffusion test and occurrence of the *blaZ* gene was moderate for *S. aureus* (kappa = 0.56) and was substantial for NAS species (kappa = 0.67), as well as for all staphylococcal species assessed together (kappa = 0.64; Table 4). Proportion of negative agreement (ranging from 0.89 to 0.94) was generally higher than proportion of positive agreement. Agreement beyond chance between *blaZ* occurrence and the nitrocefin test result was moderate (kappa = 0.62); 35.8% of the *blaZ*-positive isolates were nitrocefin-test negative, and 6.2% of the *blaZ* -negative isolates were nitrocefin-test positive. Overall, more commonly genotypically resistant isolates (those carrying *blaZ* or *mec* genes) were phenotypically susceptible than genotypically susceptible isolates (those without *blaZ*- or *mec* genes) were phenotypically resistant. Genotypic tests may be more reliable than phenotypic tests, especially when one is choosing treatment options.

**Table 4:** Agreement beyond chance between phenotypic and genotypic penicillin resistance among mastitis-causing staphylococci.

	Kappa (95% CI; *blaZ* vs. penres)	% positive agreement	% negative agreement
*S. aureus*	0.56 (0.41–0.70)	0.61	0.94
*S. chromogenes*	0.79 (0.11–1.0)	0.84	0.95
*S. epidermidis*	0.72 (0.49–0.94)	0.93	0.78
*S. haemolyticus*	0.72 (0.49–0.94)	0.83	0.89
*S. simulans*	0.45 (0.11–0.80)	0.50	0.89
Other NAS species^1^	0.18 (−0.1–0.46)	0.33	0.85
All *Staph* spp.	0.64 (0.56–0.72)	0.72	0.92

1 Includes the following species (*n*): *S. hyicus* (17), *S. xylosus* (10), *S. capitis* (9), *S. equorum* (8), *S. kloosii* (5), *M. sciuri* (5), *S. cohnii* (4), *S. pasteuri* (4), *S. hominis* (3), *S. warneri* (3), *M. lentus* (2), *S. succinus* (2), *S. auricularis* (1), *S. caprae* (1), *S. condimenti* (1), *S. gallinarum* (1), *S. pettenkoferi* (1), *S. saprophyticus* (1), and *M. vitulinus* (1).

Phenotypic penicillin resistance was assessed by disk diffusion test and genotypic resistance by occurrence of the *blaZ* gene. Agreement is considered moderate at kappa 0.41–0.6; substantial at kappa 0.61–0.8 and almost perfect at kappa 0.81–1.0 (29).

### Resistance to other antimicrobials

Resistance to other than beta-lactam antimicrobials was rare (Table 5). None of the isolates were resistant to gentamycin. Resistance to tetracycline was detectable in only six isolates (1.2%), of which three were *mecA*-positive *S. epidermidis*, one was *S. kloosii*, one *S. simulans,* and one *S. xylosus*. Resistance to enrofloxacin was detectable in three isolates (0.6%): *S. simulans,* a *mecA*-positive *S. haemolyticus*, *and* a *mecA*-positive *M. lentus*. Overall, the nine *mec*-positive isolates were the most resistant. All three ceftiofur-resistant isolates (*S. aureus*, *S. haemolyticus*, and *M. lentus*) were *mec-*gene carriers. In addition to beta-lactam resistance, three of the *mec*-positive isolates were resistant to tetracycline (all *S. epidermidis*) and two to enrofloxacin (*S. haemolyticus*, *M. lentus*).

**Table 5:** Zone inhibition diameters for enrofloxacin, gentamycin, and tetracycline among the most common staphylococcal species^1^ isolated from bovine milk.

	Inhibition zone diameters in mm; median (min – max); % resistant^2^		
Microbe^1^ (*n*)	Enrofloxacin	Gentamycin	Tetracycline
*S. aureus* (260)	29 (24–35); 0%	26 (22–38); 0%	27 (24–37); 0%
*S. chromogenes* (30)	31 (27–37); 0%	30 (26–36); 0%	29 (25–34); 0%
*S. epidermidis* (31)	35 (27–38); 0%	33 (27–42); 0%	30 (6–39); 6.4%
*S. haemolyticus* (30)	30 (6–36); 2.7%	32 (23–40); 0%	29 (22–37); 0%
*S. simulans* (57)	31 (21–36); 1.8%	31 (28–36); 0%	29 (9–35); 1.8%
Other NAS species^3^	30 (22–37); 1.6%	32 (25–41); 0%	29 (9–37); 3.1%
All isolates	0.6%	0%	1.2%

1 Species identification by MALDI-TOF MS.

2 Percentage of isolates classified as non-susceptible (including resistant and intermediate), based on disk diffusion test using EUCAST epidemiologic cut-off values when available or CLSI clinical breakpoints (see text for details).

3 Fewer than 20 isolates of each of the following species were detected (*n*): *S. hyicus* (17), *S. xylosus* (10), *S. capitis* (9), *S. equorum* (8), *S. kloosii* (5), *M. sciuri* (5), *S. cohnii* (4), *S. pasteuri* (4), *S. hominis* (3), *S. warneri* (3), *M. lentus* (2), *S. succinus* (2), *S. auricularis* (1), *S. caprae* (1), *S. condimenti* (1), *S. gallinarum* (1), *S. pettenkoferi* (1), *S. saprophyticus* (1), and *M. vitulinus* (1).

## Discussion

Our study of the antibiotic resistance of mastitis-causing staphylococci in dairy cows found that the most common form of resistance was resistance to penicillin. This is of practical importance because in Finland, penicillin is the drug of choice in mastitis treatments. When mastitis results from penicillin-resistant staphylococci, cloxacillin intramammary treatment is used (7), but antimicrobial treatment of penicillin-resistant staphylococcal mastitis, especially involving *S. aureus*, is generally not recommended because of the poor cure rate (30–32). Penicillin resistance of all isolates based on disk diffusion was 18.8%, if based on a nitrocefin test was 21.5%, but when based on *blaZ* gene carriage was higher, 26.6%. The Finnish Food Authority monitored antimicrobial resistance of staphylococci isolated from mastitic milk samples in 2005 and 2012 (15, 33). In 2005, 25% of *S. aureus* and in 2012, 23% of *S. aureus*, and 36% of NAS produced beta-lactamase, so it appears that the antimicrobial resistance of mastitis-causing staphylococci in Finland has been declining slightly.

Lower or similar rates of penicillin resistance as in the current study have been detectable in three other Nordic countries (Norway, Sweden, and Denmark). In Sweden, based on beta-lactamase production, 3% of *S. aureus* isolates and 30% of NAS isolates from clinical mastitis cases were resistant to penicillin; figures based on MIC values were similar or higher: 3% for *S. aureus* and 38% for NAS (16). In another Swedish study, 34% of NAS isolates from subclinical mastitis were beta-lactamase positive (34). In Norway, 5% of *S. aureus* and 23% of NAS isolates were penicillin resistant based on disk diffusion (12). In Denmark, 18% of *S. aureus* and 22% of NAS isolates were penicillin resistant based on MIC values (35). Penicillin resistance levels for NAS, based on MIC values, were lower in Canada, 10% (36), and in Korea, 14% (37). In some other countries, higher proportions of penicillin-resistant staphylococci isolated from bovine milk have also been detectable. In France, of almost 7,000 coagulase-positive *Staphylococcus* isolates (mainly *S. aureus*) collected during 2006–2016, 40% were penicillin resistant based on disk diffusion (38). In Brazil, 36% of *Staphylococcus* isolates from bovine mastitis, mainly *S. aureus*, were *blaZ* positive (39). In South Africa, 63% of 142 NAS isolates from bovine subclinical mastitis were penicillin resistant based on disk diffusion (40).

It is worthwhile to note, however, that a direct comparison of the results between studies is difficult, as study populations, sample collection, and sources of the isolates and methods for antimicrobial susceptibility testing differ. Another explanatory factor for differences in penicillin resistance figures may be related to the distribution of NAS species. In a Swedish study by Nyman et al. (34), *S. epidermidis* was the most common species, involving 26% of the 783 NAS isolates; similarly, in our study, *S. epidermidis* comprised about 20% of all NAS species. In contrast, in a Canadian study, where the reported penicillin resistance was lower, only 4% of over 1700 NAS isolates were *S. epidermidis*, and the three most prevalent species: *S. chromogenes*, *S. simulans*, and *S. xylosus* covered 70% of all isolates (36).

Huge differences in penicillin resistance between the *Staphylococcus* species were detectable both in ours and other studies. In our study, the lowest proportion of penicillin-resistant isolates was in *S. simulans*, and the highest in *S. epidermidis*, based on disk diffusion, nitrocefin production, and *blaZ* carriage. Many other studies have also shown *S. simulans* to be mainly susceptible to penicillin and shown *S. epidermidis* to be the staphylococcal species most resistant to penicillin and to several other antimicrobials (12, 36, 37). Multidrug resistance has also been most common in *S. epidermidis* (11, 40). In addition, *S. epidermidis* is the *Staphylococcus* species that most commonly carries the *mecA* gene coding for methicillin resistance (11, 36, 37), and consistently, of the eight *mecA-*positive isolates in the current study, six were *S. epidermidis*.

The prevalence of *mec*-positive isolates was low in *S. aureus* (0.4%), and slightly higher (3.3%) in NAS. In FINRES-Vet antimicrobial resistance monitoring in 2012, no *S. aureus* but five NAS (5.7%) carried the *mecA* gene (15). In the study by Gindonis et al. (41) which utilized three different Finnish samplings of staphylococci from bovine mastitis from previous studies, 1.5% of *S. aureus* and 1.8–5.2% of NAS isolates were *mec*-gene positive. Although in Finland methicillin resistance in mastitis-causing staphylococci is low, it may, in rare cases, cause problems in clinical work. The occurrence of the *mec* genes is not routinely tested for in mastitis cases. Some *blaZ*-negative isolates may be *mec* positive and thus penicillin resistant. The only *mecC*-positive *S. aureus* isolate in our study was *blaZ* negative and was incorrectly classified as penicillin susceptible. Penicillin treatment of these cases would have led to treatment failure.

Besides resulting in ineffective treatments, methicillin-resistant staphylococci can transfer from cows to humans. In general, although staphylococcal lineages are host-specific, host shifts may occur (42, 43). Currently, methicillin resistance of staphylococci is no problem in Finnish dairy production, but in swine production MRSA has emerged. In a year-long survey in 2016–2017, MRSA emerged in 77% of slaughter batches (17). In the same time period, MRSA infections caused by the livestock-associated CC398 have emerged in Finnish swine farmers (44). Some of the *mecA*-positive isolates in our study were also resistant to tetracycline or enrofloxacin. Although in Finland tetracycline is not the drug of choice for mastitis therapy, it is commonly used for treating other infectious diseases of cattle, especially respiratory diseases. Enrofloxacin is, among antimicrobials, the drug of choice in Finland for severe coliform mastitis.

The oxacillin disk-diffusion test better indicated *mecA* carriage than did the cefoxitin disk-diffusion test. However, many of the oxacillin disk-diffusion test results were false positive, i.e., *mec*-negative isolates showing resistance to oxacillin. Neither of the tests was, however, perfect for detecting *mec*-gene carriage. The European Committee on Antimicrobial Susceptibility Testing (EUCAST) and the Clinical and the Laboratory Standards Institute (CLSI) recommend cefoxitin disk for screening of *mecA*-mediated beta-lactam resistance in *Staphylococcus aureus* and NAS (25, 45), but in our study it did not detect potential *mecA*-positive isolates very effectively. The cefoxitin disk is reported to perform better for *mecC* screening than does the oxacillin disk (46, 47). The only *mecC*-positive *S. aureus* isolate in our study was detectable by both methods. We did not test for the recently detected *mec*-gene variant *mecB* (48), and other resistance mechanisms may also exist, such as mutations in the *gdpP* gene (49).

In Finland, veterinarians choose an antimicrobial drug for treatment of staphylococcal mastitis largely based on the *blaZ* result in the PCR test (PathoProof™ Complete-16 PCR Assay). After purchasing the equipment for analyzing milk samples with the q-rt PCR methodology, the clinical laboratory at the Department of Production Animal Medicine, University of Helsinki, analyzed milk samples both with PCR and with conventional culturing and detection of beta-lactamase production in staphylococci (Nitrocefin test, ThermoFisher Scientific). The discrepancy between these results has led to a challenge in decision-making regarding the management of an IMI case: what is the likelihood of cure and which antimicrobial to choose, if the case is treated with antimicrobials? Or is it preferable to dry off the infected quarter or even to cull the cow? The discrepancy between *blaZ*-gene carriage and the phenotypic penicillin resistance detected in our and other studies in veterinary (9, 50) and human (51, 52) medicine is an interesting phenomenon. *BlaZ*-negative but phenotypically penicillin-resistant *S. aureus* isolates are scarce (9), but *blaZ*-positive isolates that are phenotypically penicillin sensitive commonly exist (9, 50, 51). In our study, the *blaZ* gene was identified with PathoProof™ Complete-16 PCR Assay (Thermo Fisher Scientific) directly from milk, not from cultivated isolates. Therefore, we excluded from the study the samples positive for more than one staphylococcal species and the *blaZ* gene, as in samples with more than one species it was not possible to distinguish which bacterial species carried the *blaZ* gene.

Different tests typically show different sensitivities for detection of beta-lactamase production. Pitkälä et al. (53) compared six tests, using the *blaZ* PCR as the reference method. At least one method was always positive, supporting the potential for beta-lactamase production of the *blaZ*-positive isolates. Some authors report that tests based on detection of beta-lactamase production (e.g., nitrocefin test, clover leaf test) correlate better with the occurrence of the *blaZ* gene than does the agar dilution method (54). Others have reported lower sensitivities for these tests (51, 55). The results of the agar dilution and MIC methods depend on the set cut-off values. In our study, the agreement beyond chance between phenotypic penicillin resistance based on the disk diffusion test and occurrence of the *blaZ* was moderate to substantial, depending on the *Staphylococcus* species. One possible reason for phenotypically penicillin susceptible but *blaZ*-positive isolates is impaired function of the *blaZ* or its regulators, the *blaI* and *blaR* genes, because of sequence mutations (56). Whether all *blaZ*-positive isolates produce beta-lactamase *in vivo* remains to be solved. Meanwhile, *blaZ*-positive results should be interpreted as originating from potentially penicillin-resistant isolates. In practice, the possible discrepancy between occurrence of the *blaZ* gene and penicillin susceptibility may cause problems in antimicrobial treatment of mastitis.

Resistance to enrofloxacin, gentamycin, and tetracycline was rare for us, similarly to findings in most other studies. None of our isolates was resistant to gentamycin, and most of the few isolates resistant to enrofloxacin or tetracycline carried the *mecA* gene. Fergestad et al. (12) found, among 100 *Staphylococcus* isolates from clinical mastitis in Norway, only three isolates resistant to gentamycin, and three isolates resistant to tetracycline. Duse et al. (16) found no gentamycin resistance in Swedish bovine mastitis isolates. All of their NAS isolates were susceptible to enrofloxacin and tetracycline, and tetracycline resistance was also rare for *S. aureus* (16). Chehabi et al. (35) found no gentamycin resistance among 63 Danish *S. aureus* isolates, with only one isolate being tetracycline resistant. Of 49 Danish NAS isolates, one was gentamycin resistant, and 5 isolates (10%) were tetracycline resistant. The level of resistance among NAS species varies considerably. Nobrega et al. (36) reported 10% tetracycline resistance for their entire NAS group in Canada, but the percentage of resistant isolates of their most common NAS species ranged from 2% in *S. chromogenes* to 31% in *S. xylosus* and 32% in *S. epidermidis*. Among similar findings in South Korea by Kim et al. (37) were tetracycline resistance in 4% of *S. chromogenes* and in 26% of *S. epidermidis*.

## Conclusion

Our results support earlier findings that penicillin resistance is the only significant form of antimicrobial resistance among mastitis-causing staphylococci in Finland, and the proportion of resistant isolates has not increased. Based on *blaZ*-gene carriage, less than one-third of all *Staphylococcus* isolates were resistant to penicillin, and in phenotypic testing, penicillin resistance was even lower. The difference in proportions of penicillin resistance between *Staphylococcus* species, however, was considerable, lowest in *S. simulans* and highest in *S. epidermidis*. Methicillin resistance was rare, except in *S. epidermidis*, of which 12% were *mecA* positive. Some phenotypically penicillin-susceptible staphylococci carried the *blaZ* gene but isolates without *blaZ* or *mec* genes rarely exhibited resistance, suggesting that the more reliable treatment choice may depend upon genotypic AMR testing. The drug of choice for mastitis treatments in Finland is penicillin and resistance to antimicrobials other than penicillin was rare. With targeted treatment decisions, based on the knowledge on infection-causing pathogens, it is possible to keep levels of resistance to antimicrobials low.

## Data availability statement

The raw data supporting the conclusions of this article will be made available by the authors, without undue reservation.

## Author contributions

PRS planned the study. PRS and ST, and H-TT contributed to data organization and statistical analyses. ST wrote the first draft of the manuscript. All authors contributed to the article and approved the submitted version.

## Funding

The work was supported by the Walter Ehrström Foundation.

## Conflict of interest

The authors declare that the research was conducted in the absence of any commercial or financial relationships that could be construed as a potential conflict of interest.

## Publisher’s note

All claims expressed in this article are solely those of the authors and do not necessarily represent those of their affiliated organizations, or those of the publisher, the editors and the reviewers. Any product that may be evaluated in this article, or claim that may be made by its manufacturer, is not guaranteed or endorsed by the publisher.
